# The Relationship between Auditory Analysis and Dictation Skills among Jordanian Fourth-Grade Students with Learning Difficulties

**DOI:** 10.3390/children11030357

**Published:** 2024-03-18

**Authors:** Ahmad Abdelhameed Al-Makahleh

**Affiliations:** Department of Special Education, Princess Rahma University College, Al-Balqa Applied University, Salt 19117, Jordan; dr.amakahleh@bau.edu.jo

**Keywords:** auditory analysis, dictation skills, Jordanian, fourth grade, learning difficulties

## Abstract

This study aims to explore the skills of Jordanian Arabic-speaking fourth graders with learning difficulties in terms of auditory analysis and dictation tests. It mainly aims to investigate the relationship between students’ abilities to perform auditory analyses and dictation tests. The sample in the study consists of 110 Jordanian fourth graders, who are then divided into 54 students with learning difficulties who are diagnosed as having a satisfactory level of reading and writing and 56 typically developing students. The students are asked to respond to two tests, the auditory analysis and the phonological awareness test, which are prepared by the researcher himself. The results demonstrate a statistically significant positive correlation between the auditory analysis and dictation skills of fourth-grade students with learning difficulties. This means that improving the auditory analysis skills corresponds to an increase in the dictation skills of these students. The results also reveal a statistically significant correlation between auditory analysis and dictation skills in typically developing students.

## 1. Introduction

The term “learning difficulties” (henceforth, LDs) is historically known to refer to children who experience difficulties to reading, writing, or math skills; however, they are not struggling with developmental disabilities. According to the National Advisory Committee on Disability Affairs in America’s report, a LD is “a disorder in one or more of the basic psychological processes necessary for either understanding or using spoken or written language”. It manifests as a deficit in mathematical operations, dictation, reading, writing, speaking, listening, or thinking. Cases of brain damage, cognitive impairments, brain malfunction, dyslexia, or aphasia are also included in this phrase. Children with LDs resulting from mental retardation, inadequate social–cultural functioning, or problems with vision, hearing, movement, or emotions are excluded from this category [1]. The definitions of LDs generally focus on the criteria for identifying LDs, such as contrast and exclusion, in addition to an obvious weakness in cognitive processes, auditory awareness, and visual awareness, which greatly affects the learning of academic skills in the Arabic language (reading and writing) and mathematics. It can also lead to problems with language or thinking.

The classification of an LD varies across researchers and depends on their field interest and treatment intervention [2]. One of the most modern methods of classifying and identifying children with LDs is the response-to-intervention (RTI) approach, which aims to deliver appropriate educational services within an inclusive framework. The three phases of the intervention start with administering screening tests to identify at-risk children and then providing gradual support services [3]. However, screening the level of auditory analysis and dictation skills as early as possible facilitates meeting the needs of students with LDs by providing multi-phase RTI.

Auditory analysis is a key factor in literacy and language development, especially for students with learning difficulties [2]. It involves recognizing phonemes and manipulating sounds within words, which help in developing phonological awareness, which is an essential component of mastering reading and writing. Students with LDs may suffer from problems with auditory processing that profoundly impact their academic performance, particularly in terms of literacy. There is a strong correlation between cognitive processing ability and academic skills, that is, the auditory and visual cognitive abilities affect various learning skills, such as reading, writing, and arithmetic. Writing skills require various integrated abilities, including visual–motor skills, memory, analysis, and visual discrimination. If a student has difficulty processing these abilities, he/she will then suffer difficulties in relation to dictation and writing skills [4].

Writing is one of the basic skills in the learning process and it has different forms, such as guided writing, dictation activities, and composition. Each of these has different requirements. Writing, further, is the process of recognizing and using letters, figures, and visual and tactile symbols that indicate sound according to cognitive processing. These symbols express the ideas intended to be conveyed to others [5].

Dictation, on the other hand, is based on three basic skills. First, identifying a word orally to determine the sounds that make it up. Second, hearing a letter and distinguishing it from other letters. Third, recalling the shapes of the letters based on their position in the word, especially in languages where each letter has various shapes, such as in Arabic. Struggling with these skills mainly leads to failure in terms of dictation. The conflation of various letters stems from a deficiency in visually distinguishing between their written forms, accompanied by a weak ability to discern the correspondence between the letters’ sounds and their visual representations. This challenge appears in sounds that closely resemble each other, especially those that have the same place of articulation, and it extends to the phonetic structure of words, which relies on auditory analysis and phonemic awareness. To offer an example, someone might mistakenly say *wardˤa* (a nonsense word in the Arabic language) instead of *warda* (which means “a rose” in the Arabic language) [6].

Dictation difficulties have become worse among students with learning difficulties who struggle with their academic performance. Although this group is characterized by average intelligence, they encounter challenges in relation to certain learning processes, distinct from those with mental or sensory disabilities or multiple disabilities. It is noteworthy that individuals with emotional disorders, coupled with brain dysfunction, have a reduced ability to follow-up academically with their peers. They exhibit deficiencies in memory processes, attention, perception, and basic skills [7].

Furthermore, auditory analysis is identified as processing, understanding, and analyzing sounds. It is a basic skill for reading and dictation. Children who experience difficulty with auditory discrimination struggle to develop phonological awareness, knowing that sound auditory analysis does not guarantee the development of the phonological awareness of sounds.

Writing is closely linked to audio analysis skills in that auditory analysis skills contribute significantly to understanding syntactic rules and sentence structure. Therefore, writing skills are frequently affected because students with language difficulties may struggle to express their thoughts meaningfully, maintain grammatical precision, and adhere to established syntactical structures. These obstacles contribute to limiting their ability to express themselves effectively within various written assignments, essays, and academic tasks [8].

### 1.1. The Statement of the Problem

Auditory analysis skills and dictation skills are essential components of literacy development for all students. They need to acquire these skills to be able to read and write independently. Students may face difficulties in terms of writing skills due to their weak ability to perform sound auditory analysis. Dictation difficulties often stem from phonological processing difficulties, which make it difficult to link sounds to letters and words correctly. This may be more challenging for students with LDs where they experience an inability to convey meaningful ideas in writing or understanding words and sentences in reading. Therefore, this study examines the relationship between auditory analysis skills and dictation skills among fourth-grade students.

### 1.2. Questions of the Study

Is there a statistically significant correlation between auditory analysis skills and dictation skills among Jordanian fourth-grade students with LDs?

Is there a statistically significant correlation between auditory analysis skills and dictation skills among Jordanian typically developing fourth-grade students?

Are there any statistically significant differences in auditory analysis skills and dictation skills between students with LDs and their typically developing peers in the fourth grade?

### 1.3. Theoretical Framework

This section aims to present the theoretical framework concerning auditory analysis, dictation, and writing skills.

#### 1.3.1. Auditory Analysis

Auditory analysis involves processing, understanding, and analyzing sounds. It is a basic skill for reading and dictation. Children who have difficulty in terms of auditory discrimination struggle to develop phonological awareness, knowing that sound auditory analysis does not guarantee the development of sound phonological awareness.

The auditory analysis skill is the student’s ability to recognize the entire word in case of missing one or more letters. It is considered a skill that makes up the auditory awareness through which the student can convey the meaning and interpret the auditory stimuli he/she hears [9]. A child with difficulties to auditory awareness commonly has difficulty understanding the sounds he/she hears and confuses letters that are similar in sound, e.g., (e, i), and words, e.g., (pen, pin), which affects the child’s understanding and ability to communicate with others.

Evaluating the auditory analysis process is based on recognizing sounds in spoken words, identifying the student’s readiness to learn reading and dictation, and evaluating deficiencies in this area. It also provides diagnostic information that specialists can use to provide support and adequate intervention [10]. The significance of auditory analysis stems from its ability to identify deficiencies in hearing and auditory awareness, particularly in individuals with learning disabilities. These deficiencies can be found in learning skills, including reading and dictation, and they are typically more prevalent in learning-disabled children than in typically developing children. The issues related to auditory analysis can be categorized into four areas: auditory awareness, auditory memory, auditory closure, and auditory analysis. These issues can also be identified by observing auditory behavior and using screening tests designed for this purpose [11].

The evaluation of auditory analysis skills involves the examination of both simple and complex words. This evaluation entails the removal of a specific part from a word, leaving behind a segment that still constitutes a meaningful word. The examiner says the word and then instructs the child to pronounce the remaining portion after the designated deletion. The word selection for this test considers variations in phonetic syllables, ensuring that each word retains a meaningful component when a specific part is removed, whether at the beginning, middle, or end. The test aims to gauge the child’s capacity to deconstruct sound patterns into their constituent elements and recognize the resultant sound pattern when a specific part of the original is omitted. The test also seeks to identify any shortcomings in phonetic analysis skills and their potential implications for academic performance, particularly in terms of reading and dictation [12].

#### 1.3.2. Dictation Skill

Dictation is regarded as one of the most substantial skills that a student must master in the early academic stages because it has a significant impact on the academic development process. It is a skill that requires the development of mental and physical abilities. It is known as the symbolic tool that embodies the sounds of speech in the form of visual symbols that express the words and take into account the known rules of dictation. Writing also means the ability to form the shapes of letters correctly and easily [10].

Learning to spell includes a child’s mastery of the single-handed skill, dexterity with a pencil, alignment, direction awareness, differentiation between concrete and abstract objects, directed visual attention to particular stimuli, replication of basic geometric shapes, replication of letters in the same context, and replication of letters from different sources. This skill is demonstrated by distinguishing well-made letters from poorly made ones on a chalkboard or piece of paper, accurately duplicating letters with comparable shapes, maintaining acceptable word and letter spacing, and connecting letters appropriately [13].

Dictation is one of the productive abilities that kids acquire after linguistic input, which makes it important to the learning process. Learning to write is more than just language and expression; it also involves formulation, composition, and analysis. Therefore, teachers in educational settings are responsible for ensuring students’ writing skills are developed, especially in the early stages [12].

Learners face various dictation and writing difficulties, which lead to poor writing performance. According to [14], these challenges are primarily caused by deficiencies in attention and mental focus, difficulties with motor execution, inaccurate visualizing or re-conceptualizing words and letters, and a decrease in the retention of motor and rhythmic patterns related to writing letters and words. This variety of contributing factors has been divided into two primary categories: first, intrinsic factors, which include problems such as poor motor control, poor spatial perception, and poor visual acuity; and second, extrinsic factors, which result from inadequate instruction and an inappropriate learning environment [15].

As for the difficulty related to learning to write in particular, Al-Batayneh idefined it as a difficulty or incapacity to see letters or words [16]. This indicates that in addition to poor handwriting organization or inability to express things successfully, confusion between letters is also one of the symptoms of dysgraphia. The types of difficulties related to writing vary according to several considerations. Furthermore, Al-zayat identified four types of dysgraphia: inconsistency of letters, confusion between letters that are similar in shape or sound, irregular shape and size of letters, and incomplete writing of a letter or word [17]. It can be said that these difficulties are related to the mental abilities of perception, attention, concentration, and organization, in addition to motor abilities, such as holding a pen, drawing letters, and others.

Regarding the difficulty related to learning to write in particular, it indicates a difficulty or inability to visualize letter shapes and words, which is considered one of the manifestations of dysgraphia, in addition to poor handwriting or inability to express thoughts in written form [16] Al-Batayneh. Moreover, [17] Al-Zayat identified four types of dysgraphia: inconsistency in letters, confusion between letters that are similar in shape or sound, irregular shape and size, and incomplete writing of a letter or word. These difficulties may be related to mental abilities (perception, attention, concentration, and organization) and motor abilities, such as holding a pen, forming letters, etc.

Productive writing for students with hearing impairment requires integrating word recognition, comprehension, and writing skills. For example, analyzing the symbols of visible words is considered one of the important processes that achieve comprehensive word recognition. Likewise, written comprehension requires experience, and a conclusion is achieved through understanding ideas and their sequence. Thus, writing programs for students with hearing impairment should focus on extracting ideas from the written text and practicing writing words as an activity, considering the student’s developmental level and interests [18].

Students with learning difficulties commonly exhibit a limited vocabulary and tend to punctuate their writing in a manner comparable to their typical peers. When writing, these students encounter difficulties in adhering to proper rules of grammar. The challenges manifest in omitting subjects and verbs, neglecting pronoun references, excluding inflectional endings, and grappling with incorporating intricate phrases. In addition to contending with redundant and extraneous language issues, these students often contend with omissions of crucial words, misplacement of words within sentences, struggles in employing advanced writing techniques, and a propensity for frequent dictation errors [12].

Al-Batayneh outlined the distinctive attributes that characterize the writing of students with learning difficulties [16]. These features encompass inaccurate transcription, a protracted duration required to complete written assignments, a tendency to separate connected letters within words, maintaining the proximity of the eyes to the page during writing, incorrect pen grip, inconsistencies in letter formation, sporadic mixing of uppercase and lowercase letters, and frequent letter reversals. Furthermore, slow processing of oral or written language, difficulties in extracting ideas from the text, delayed word recall, difficulties in understanding language usage rules, distortion of letter images during writing, errors in organizing words within a sentence, difficulties in interpreting and composing sentences, difficulties in filling in sentence blanks, poor organization of sentences and paragraphs, carelessness in reviewing written work, an inability to correct errors, and writing incoherent sentences.

Al-Hawari described the unique traits of a child with a learning difficulty when it comes to writing, emphasizing how these traits differ from those of a child developing normally in several areas [19]. Notably, problems with language comprehension emerge, as demonstrated by the child’s inability to comprehend instructions, apparent inattention, difficulties understanding abstract terms, and uncertainty about time. A child with expressive language issues may be reluctant to answer questions or participate in discussions, their vocabulary may be less, and their speech may occasionally be disordered. [19] Al-Hawari also emphasized that receptive and expressive language issues greatly impact the writing process since they heavily rely on awareness, auditory analysis, and phonetic awareness. 

### 1.4. Review of Related Literature

In the USA, Arfé and Perondi examined students with learning difficulties in terms of their writing and dictation patterns compared to their typical peers using an applied model for written stories [20]. The study sample consisted of 34 students, and half of them had learning difficulties. It was observed that students with learning difficulties used the same method as the typically developing students in listening and writing, but they were characterized by repeating words and names in terms of shape only when writing. The results also indicated that students with learning difficulties have problems with writing strategies and delays in linguistic experiences.

Moreover, Al-Hayek and El-Zraigat investigated the effectiveness of a training program in addressing the difficulties when writing compositions among hearing-impaired students in Jordan using a quasi-experimental approach [21]. The sample in the study consisted of 52 sixth graders who were then divided into two groups: the experimental group consisted of 24 students who were trained using the written composition program, and the control group consisted of 28 students who were trained using the traditional methods. It was found that students with hearing impairment struggle with various difficulties and that their skill in terms of writing compositions is weak in the areas of form and content; however, they face more challenges in content. The training program effectively addressed the composition difficulties facing students with hearing impairment. The study recommended paying more attention to the functional writing skills of students with hearing impairment and enriching the curricula with writing activities. 

Furthermore, Ref. [22] conducted a study to reveal the effectiveness of an auditory awareness development program in improving expressive language skills in children with central auditory processing disorder (CAPD). The study sample consisted of 16 children with auditory processing disorder, where 10 students aged between 6 and 9 years obtained low scores on the auditory awareness and expressive language skills scales and were classified as having average intelligence. Several tools were used: the Auditory Processing Disorder Diagnostic Scale, Auditory Perception Scale, Expressive Language Scale, and Auditory Perception Development Program, all prepared by the researcher, and the Color Progressive Matrices Test (John Raven). It was found that the program achieved positive results in developing auditory awareness among the participating children. The group’s performance on the Expressive Language Scale improved after implementing the program. The program’s positive impact has continued; the group’s performance on the auditory awareness and expressive language scales has not changed over one month and a half. 

On the other hand, Ref. [23] attempted to reveal the effectiveness of a behavioral–motor training program in developing sensory–motor synergy and visual memory among students with learning difficulties in terms of writing. The researcher designed a training program that was delivered to a sample of 20 students who struggle with writing, accompanied by difficulties in sensory–motor coordination and visual memory. A quasi-experimental design based on one group was relied upon, with a pre- and post-measurement. The researcher used several tools, including an exploratory study, in the diagnosis and nomination stage (interviews, observation network, sensory–motor synergy rating scale, visual memory rating scale—directed at teachers—and the “Raven” intelligence test). Other tools were employed during the study such as a writing test, a visual memory test, and a measure of sensory–motor synergy.

The program was delivered to the experimental sample in a total of twenty-two sessions, two sessions per week over three months. The proposed training program effectively developed sensory–motor synergy and visual memory in students with writing difficulties. Differences between the pre- and post-measurements were observed on the sensory–motor synergy scale and between the pre- and post-measurements in the averages of the sample members’ scores on the visual memory test.

According to what has been mentioned above, the previous studies helped the researcher to support and enrich the study’s theoretical framework and develop the study’s tool. By comparing the results of previous studies with the current study, the researcher observed that some studies dealt with writing skills, whereas some examined writing based on a model and its relationship to visual–motor synergy and identified the nature of the dictation errors among students in schools [4,24]. Moreover, some studies have examined the effectiveness of a program in improving the reading process, and most of these studies recruited samples of students without learning difficulties [22]. This study is distinguished from previous studies in that it was conducted to identify the relationship between the skills in terms of auditory analysis and dictation among students with learning difficulties compared to typically developing students.

## 2. Materials and Methods

This section aims to present the approach, the sample, and the procedure of the current study.

### 2.1. Approach

A descriptive and correlational approach was employed in this study since it is an appropriate method to identify auditory analysis and its relationship to dictation among fourth-grade students with LDs, using observation cards as a tool.

### 2.2. Population and Sample

The population of the current study comprises Jordanian Arabic-speaking fourth graders. The decision to select fourth-grade students was based on the outcomes of Jordanian curricula. In this educational stage, students should be able to read and write independently. However, students with LDs normally receive an official diagnosis in grade two or three, and this late diagnosis hinders them from receiving early intervention services. The diagnostic process begins when teachers refer students to the school assessment team, who administer formal tools that are prepared by the Ministry of Education. 

The sample in the study comprised a total of 110 male and female fourth-grade students. All the participants were enrolled in Salt Educational Public Schools in Jordan in the academic year 2022/2023. The students were divided into two groups: the first group consisted of 54 fourth graders who were diagnosed with mild LDs based on their official diagnosis statement. On the other hand, the second group consisted of 56 typically developing students.

### 2.3. Instruments

The researcher developed two tests to measure the level of auditory analysis and dictation skills of the fourth graders based on the following steps. First, to determine the goal of the test, which is to measure the level of auditory analysis or the dictation skills. Second, to prepare the initial version of the study tool (tests for auditory analysis and dictation). 

The study’s instruments included an auditory analysis test and a dictation test. The items in these tests were informed by the teacher’s guidebook, which includes dictation activities suitable for the fourth grade and instructions for using these tests. The content of the instruments was refined by a panel of 10 academics experienced in curricula and teaching methods in Jordanian universities, and it was modified according to their suggestions. 

The auditory analysis test consisted of five questions, and each question included five sub-items that suit the nature of phonetics in the Arabic language. Each question was developed to ask about one type of word. The types of words were three-root words with short vowels, words with the long vowel /a:/, words with the long vowel /u:/, words with the long vowel /i:/, and finally, different words with consonant syllables. For each type of word, the students were asked about four words. Each question aimed to measure the student’s ability to perform the auditory analysis. It aimed specifically to measure the student’s ability to recognize the word after deleting one of its consonants or syllables. Each student was tested individually by his/her teacher. The teacher read the examined words to each student and asked them, “What is the expected word that we have if we delete, e.g., a consonant or a syllable?” To offer an example, the teacher asked the student, “What is the expected word that we have if we delete the syllable /qa/ from the word /qalam/, which means “a pencil”. The student received one mark if he knew the correct word and zero if he did not. 

The dictation test also consisted of five questions, and each question was concerned with one type of word: the three-root words with short vowels, words with a long vowel /a:/, words with a long vowel /u:/, words with a long vowel /i:/, and finally, different structured words. There were ten words under each type of word. Each student was asked verbally to write down four words (chosen randomly by their teacher), one word at a time, for each type of word. This meant that each student had to write down 20 words; four words for each word type. The test was administered by the teacher for each student individually. The student received a mark for each correct word and a zero for each incorrect word.

#### 2.3.1. Validity of the Instruments

To validate the instruments used in the study, the auditory analysis and dictation tests were administered to a pilot sample of 25 students with LDs in the fourth grade. The decision to exclude typically developing students from the pilot sample was based on the assumption that if the items in the study instruments are clear and understandable for students with LDs, the items will be appropriate for typically developing peers. Piloting of the instruments was performed by the researcher, which enabled him to refine the items. Table 1 presents the values of the correlation coefficients of the auditory analysis and the dictation tests that were administered to a pilot sample of 25 students with learning difficulties.

Table 1 shows that the correlation coefficient values between the items in the auditory analysis test and the total score ranged from 0.725 to 0.946. Furthermore, the values of the correlation coefficients for the dictation test’s items and the total score ranged from 0.809 to 0.965. These values are considered high and acceptable, demonstrating the construct validity of the study’s tests.

#### 2.3.2. Difficulty and Discrimination Coefficients

Table 2 presents the difficulty and discrimination coefficients for the performance of the students with learning difficulties (n = 25) on the auditory analysis and the dictation tests. 

Table 2 shows that the difficulty coefficients of the performance of the students in the auditory analysis ranged from 0.34 to 0.79, while the discrimination coefficients ranged between 0.602 and 0.890 for the same test. The difficulty score is more difficult when it is closer to zero, and vice versa. Furthermore, Table 2 shows that the difficulty coefficients of the performance of the students in the dictation test ranged between 0.28 and 0.60, and the discrimination coefficients ranged from 0.724 to 0.937. These values are acceptable for the difficulty and discrimination coefficients.

#### 2.3.3. The Reliability of the Tests

To check the reliability of the auditory and dictation tests, they were administered to a pilot sample of 25 students with learning difficulties from grade four using the internal consistency method involving Cronbach’s alpha equation. The results revealed acceptable values, indicating the good reliability of the auditory test, i.e., 0.883 and the dictation test, i.e., 0.943.

## 3. Results and Discussion

This study attempts to investigate the relationship between audio analysis and dictation skills among Arabic-speaking fourth-grade students enrolled in governmental schools in Jordan. To answer the first two questions, the Pearson correlation coefficient test was used to test the collected data. This statistic test is considered appropriate to measure the association between the variables, as the variables in the current study are continuous [24]. To answer the third question, the means, standard deviations, and *t*-test scores were calculated.

### 3.1. Results and Discussion of the First Question 

This section aims to answer the first question: “Is there a statistically significant correlation between the auditory analysis ability and dictation skills among fourth-grade students with LDs?”

Therefore, to see whether there is any statistically significant correlation in the performance of the fourth-grade students with learning difficulties in the auditory analysis ability and dictation skills, the Pearson correlation coefficient was calculated. Table 3 shows the results of the Pearson correlation coefficient between the auditory analysis and dictation skills in the performance of fourth-grade students with learning difficulties.

Table 3 reveals a positive and statistically significant correlation between the performance of fourth-grade students with learning difficulties in the auditory analysis test and dictation test. That is, the value of the correlation coefficient is 0.567 and it is statistically significant at 0.01. This positive correlation suggests that a boost in auditory analysis skills corresponds significantly to improved dictation skills among fourth-grade students with learning difficulties. However, a decline in the auditory analysis ability results in a decrease in the dictation skill among fourth-grade students with learning difficulties.

The researcher attributes the aforementioned results to the fact that good training in the phonetic analysis of syllables and words develops relevant skills, creates awareness of the words dictated, and matches the word’s pronunciation to its form. This indicates that the ability of students with learning difficulties to perceive letters, syllables, and pronounced words phonetically increases their ability to spell words and overcome the difficulties they face in dictation. The results are also attributed to students’ challenges in terms of dictating words correctly. This challenge stems from a deficiency in the auditory analysis of words during the dictation exercises in the Arabic language, especially for the dictation of Tanween (Nounation), the/h/sound, written but not pronounced sounds, and pronounced but not written sounds. Consequently, these difficulties resulted in a deficiency in dictation skills among the students with learning difficulties.

These findings are in line with [20,21,22,23], which suggested that students with learning difficulties had poor performance in writing tests, especially in dictation, because of their deficiency in auditory analysis and phonological awareness.

### 3.2. Results and Discussion of the Second Question

This section aims to answer the second question: “Is there a statistically significant correlation between the performance of the typically developing fourth graders in the auditory analysis and dictation tests?”

Therefore, to see whether there is any statistically significant correlation in the performance of the typically developing fourth graders in the auditory analysis ability and dictation skills, the Pearson correlation coefficient was calculated. Table 4 presents the results of the Pearson correlation coefficient between the auditory analysis and dictation skills in the performance of the typically developing fourth graders.

Table 4 reveals a statistically significant positive correlation between the ability in terms of auditory analysis and dictation skills in the performance of typically developing fourth graders. That is, the correlation coefficient is 0.410, indicating a statistically significant value at the significance level of 0.05. The positive correlation indicated that an enhancement of the auditory analysis ability corresponds to an increase in dictation skills among fourth-grade students without learning difficulties, and a decrease in auditory analysis ability leads to a decrease in dictation skills among them. This result supported the fact that auditory analysis ability and phonological awareness have an important role in developing the ability of typically developing children to spell words correctly and develop writing skills in general.

These findings supported the results of [21,22,23], which suggested a positive correlation between the improvement of phonological awareness and auditory skills and the improvement of writing skills in fourth graders.

### 3.3. Results and Discussion of the Third Question

This section aims to answer the third question: “Are there any statistically significant differences in auditory analysis skills and dictation skills between the students with LDs and their typically developing peers in fourth grade?”

To answer this question, Table 5 presents the means (Ms) and standard deviations (SDs) of the performance of typically developing fourth graders and their peers with learning difficulties in the auditory analysis and dictation tests. Furthermore, Table 5 shows the results of an independent *t*-test to determine the differences between the performances of the two groups of children in both tests. 

Table 5 reveals that the means and standard deviations of the performances of typically developing children and their peers with learning difficulties on the auditory analysis test are M = 19.36; SD = 0.989 and M = 8.70; SD = 2.515, respectively. It also shows that the performances of the two groups of children are statistically significant, that is, t-value = (−29.466 ≤ 0.05). This result demonstrates statistically significant differences between the performance of typically developing students and students with learning difficulties in favor of the typically developing students.

On the other hand, Table 5 shows that the means and standard deviations of the performances on the dictation test of typically developing children and their peers with learning difficulties are M= 19.71; SD = 0.530 and M = 4.89; SD = 1.538, respectively. It also shows that the performances of the two groups of children are statistically significant, that is, t-value = (−68.088 ≤ 0.05). This finding suggests significant differences in the dictation test between students with learning difficulties and typically developing students in grade four in favor of the latter. This can be attributed to the fact that students with LDs have several characteristics that affect their ability to read and write. Examples of these characteristics are poor attention, slow reading progress, memory difficulties, problems in conceptualization, and difficulties in soft motor skills [2].

These findings support the results of [20,21,22,23], which indicated that typically developing students had better performance than students with learning difficulties, where the latter had more serious difficulties in the writing test.

## 4. Conclusions and Recommendations

This study attempts to investigate the relationship between performance in the auditory analysis and the dictation tests among 110 Jordanian Arabic-speaking fourth graders who were divided into 56 students with learning difficulties and 54 typically developing students. The students were asked to respond to two tests, the auditory analysis and the phonological awareness tests, which were prepared by the researcher himself. The results revealed a statistically significant positive correlation between the auditory analysis and the dictation skills in fourth-grade students with learning difficulties as well as in typically developing students. However, the performance of typically developing students was better and more advanced.

Based on the results of this study, the researcher recommends holding training programs to prepare teachers of students with learning difficulties to enhance the auditory analysis skills among this group of students and to recognize their learning characteristics. The researcher also hopes that the Ministry of Education will adopt an integration strategy for students with learning difficulties, considering that this step will enhance students’ academic achievements, including their dictation skills, as this will aid collaboration and support between typically developing students and their peers with special needs.

In a general sense, the results of the current study highlighted the need for appropriate interventions and preparation of qualified professional teachers who can provide remedial programs for students with LDs. Furthermore, it is highly important to screen reading and writing skills as early as possible, which may facilitate the delivery of early intervention programs.

## Figures and Tables

**Table 1:** Correlation coefficients of the performance of the pilot sample in the auditory analysis and dictation tests.

Auditory Analysis Test		Dictation Test	
Correlation Coefficient		Correlation Coefficient	
N	Total Score	N	Total Score
1	0.725 **	1	0.809 **
2	0.791 **	2	0.947 **
3	0.830 **	3	0.876 **
4	0.857 **	4	0.932 **
5	0.946 **	5	0.965 **

** Statistically significant at 0.01.

**Table 2:** Difficulty and discrimination coefficients of the performance of the pilot sample in the auditory analysis and dictation tests.

Auditory Analysis Test			Dictation Test		
N	Difficulty	Discrimination	N	Difficulty	Discrimination
1	0.79	0.602	1	0.60	0.724
2	0.64	0.712	2	0.51	0.921
3	0.60	0.717	3	0.42	0.804
4	0.57	0.769	4	0.40	0.887
5	0.34	0.890	5	0.28	0.937

**Table 3:** Results of the Pearson correlation coefficient between the auditory analysis and dictation tests in the performance of fourth-grade students with learning difficulties.

Type of Test		Dictation
Auditory analysis	Correlation coefficient	0.567 **
	*p*-value	0.000

** Statistically significant at 0.01.

**Table 4:** Results of the Pearson correlation coefficient between the auditory analysis and dictation tests in the performance of typically developing fourth graders.

Type of Test		Dictation
Auditory analysis	Correlation coefficient	0.410 *
	*p*-value	0.002

* Statistically significant at 0.01.

**Table 5:** Results of an independent *t*-test of the performance of typically developing fourth graders and their peers with learning difficulties in the auditory analysis and dictation tests.

Type of Test	Category	N	Mean	SD	T-Value	Degree of Freedom	*p* Value
Auditory analysis	Students with LD	54	8.70	2.515	−29.466	108	0.000 *
	Typically developing students	56	19.36	0.989			
Dictation	Students with LD	54	4.89	1.538	−68.088	108	0.000 *
	Typically developing students	56	19.71	0.530			

* Statistically significant at 0.01.

## Data Availability

Data are available from the corresponding author upon reasonable request because the author may use the data in his upcoming research.
